# Determining the Status Quo of Infection Prevention and Control Standards in the Hospitals of Iran: A Case Study in 23 Hospitals

**DOI:** 10.5812/ircmj.14695

**Published:** 2014-02-05

**Authors:** Jalil Shojaee, Mahmood Moosazadeh

**Affiliations:** 1 Health Deputy, Mazandaran University of Medical Sciences, Sari, IR Iran; 2 Department of Epidemiology, Research Center for Modeling in Health, Institute of Future Studies in Health, Kerman University of Medical Sciences, Kerman, IR Iran

**Keywords:** Infection, Prevention, Hospital, Accreditation

## Abstract

**Background:**

Applying Prevention and Control of Infection (PCI) standards in hospitals reduces probable risks to patients, staff and visitors; it also increases efficiency, and ultimately improves productivity of hospitals.

**Objective:**

The current study aimed to determine the status quo of international standards of PCI in hospitals located in the north of Iran.

**Materials and Methods:**

This cross-sectional study was conducted in 23 hospitals. Data collection tool was a questionnaire with confirmed validity and reliability. . In this regard, 260 managers, section supervisors and infection control nurses participated in the study according to census basis. SPSS software version 16 was employed to analyze the data through descriptive and analytical statistics.

**Results:**

Among the studied hospitals, 18 hospitals were public. Hospitals enjoyed 77.2% of leadership and programming, 80.8% of focus of programs, 67.4% of isolating methods, 88.2% of hand health and protection techniques, 78.8% of improving patient’s safety and quality, 90.3% of training personnel, and 78.7% of the average status quo of PCI standards.

**Conclusions:**

This study revealed that PCI standards were significantly observed in the studied hospitals and that there were necessary conditions for full deployment of nosocomial infection surveillance.

## 1. Background

Nosocomial infections are referred to the infections that appear at least 48 to 72 hours after patient’s admission, during hospitalization or after discharge, provided that they don’t exist or incubate at the time of admission (1-4). Reports indicate that only 5-10% of hospitalized patients in industrial developed countries suffer from nosocomial infections, while the rate is 25-30% in developing countries. According to an estimation, annual incidence of nosocomial infections was about 10% (about 2 million) in the United States in 2011 (5-9). It was estimated that the average incidence of nosocomial infections in Iran in 2009 was 10-15%; 10-20% of patients with nosocomial infections died. Moreover, 5.96% of all deaths in hospitals result from nosocomial infections (10). According to the conducted case studies , it has been proved that the level of nosocomial infections is high in Iran; for example, Masoumi Asl et al. (2009) reported that the incidence of nosocomial infections in pediatric intensive care units was 14.7% (11). In a burn center with 182 hospitalized patients, the frequency of nosocomial infections was 75.9% (140 patients) (12). In addition, the incidence of urinary tract infection in 428 patients admitted to pediatric intensive care unit of Tehran Mofid Hospital was 7.2% (13).One of the methods to improve quality and safety in health care organizations is through accreditation which emphasizes the continuous improvement of quality and safety of patients and personnel in order to describe quality of health-treatment services (14).

Joint Commission International (JCI) has presented international standards for hospitals to improve their quality, and patients’ safety. These standards are a set of various initiatives designed to evaluate health care. They contain two parts: “patient-based standards” and “organization-based standards”; Prevention and Control of Infection (PCI) standards are of organization-based standards (14-19). Applying PCI standards in hospitals reduces probable risks to patients, staff and visitors, it also increases efficiency, and ultimately improves productivity of hospitals. Several studies have indicated (15-18) that accreditation standards are effective in reducing hospital infections (15-18). For example, a study conducted in Japan on 638 hospitals in 2004-2005 showed that accreditation of hospitals resulted in positive performance in infection control programs (18). Nosocomial infections increase hospital readmissions, average length of stay, costs and mortality rate; they also decrease productivity and have adverse and negative effects on the reputation of the hospital.

## 2. Objectives

The current study aimed to assess the status quo of PCI international standards in 23 hospitals in the north of Iran (Mazandaran province).

## 3. Materials and Methods

This cross-sectional study was conducted in all general and specialized public hospitals in the north of Iran in 2012-2013 (23 hospitals in Mazandaran province). Data was collected through a questionnaire completed by 260 managers, supervisors of different sections and infection control nurses according to a census base. A standard questionnaire consisted of three parts was employed to collect the data: The first part consisted of variables related to the status of hospital (name of hospital, type of hospital in terms of being general or specialized and number of beds); the second part included respondents’ demographic variables (gender, age, education and work experience); and the third part consisted of 6 sections leadership and programming (14 questions), focus of programs (35 questions), isolating methods (5 questions), hand health and protection techniques (5 questions), improving patients` safety and quality (14 questions), and training personnel (7 questions). Content Validity of the questionnaire was assessed by experts` viewpoints. To determine the reliability of the questionnaire, test-retest method was employed, that is, the final questionnaire was distributed among 10 members of the statistical universe twice with a time interval of 10 days, and its reliability was found to be 81%.

Questions of the third part had three options (Yes, somewhat and no). Each positive, somewhat, and negative answer received 2, 1, and 0 scores, respectively. Since each field had a certain number of questions, scores of each field were changed to a 0-100 scale; the average scores related to each area were measured. Scores over 75% were considered as excellent, 61-75% as good, 41-60% as medium, 25-40% as bad and less than 25% as very bad. To analyze the data analytical, and descriptive statistical methods were applied, and ANOVA, , independent T-test, Kruskal-Wallis, and Mann-Whitney tests were used. Before the analysis, normality of data and equality of variances were examined; in case that conditions of parametric tests were not established, non-parametric tests were used. SPSS software version 16 was employed to analyze the data, and P ≤ 0.05 was considered as the level of significance. The current study was conducted in coordination with the treatment deputy, the deputy sent letters to the hospitals, and the informed written consent was obtained from the respondents before completing the questionnaires. In addition, participants were informed that the analysis would not be conducted in terms of hospital name and that the name of hospitals and the respondents’ profiles would be kept confidential.

## 4. Results

In the current study, 260 participants including managers, supervisors and infection control nurses participated; 81.2% of them were male. Most of the participants were in the age group of 30-40 years, 82.7% of them had bachelor’s degrees, 86.1% of the subjects were supervisors of different sections, and 88.8% of them had studied nursing (Table 1). Among the 23 hospitals whose staff completed the questionnaires, 18 were general hospitals; most of the hospitals (12 ) had between 100 - 200 beds (Table 2). 

**Table 1.** Subjects’ Demographic Characteristics

Variables	No. (%)
**Gender**	
Male	211 (81.2)
Female	49 (18.8)
**Age group**	
20-30	35 (13.5)
30-40	112 (43.1)
40-50	99 (38.1)
< 50	14 (5.4)
**Education**	
Bachelor’s degree	215 (82.7)
Master’s degree and higher	45 (17.3)
**Participants’ position**	
Manager	13 (5)
Section in-charge	224 (86.1)
**Academic field**	
Infection control expert	23 (8.8)
Medicine	11 (4.2)
Nursing	231 (88.8)
Others	18 (6.9)
**Work record(year)**	
< 6	45 (17.3)
6-10	46 (17.7)
11-15	58 (22.3)
16-20	58 (22.3)
> 20	53 (20.4)

**Table 2.** General Characteristics of Studied Hospitals

Variables	No.	No. of Personnel Participating in the Study (%)
**Type of hospital**		
General	18	210 (80.8)
specialized	5	50 (19.2)
**No. of beds**		
< 99	9	78 (30)
100-200	12	161 (61.9)
> 200	2	21 (8.1)
**Total**	23	260 (100)

Table 3 indicates that the differences observed in all components of PCI (leadership and programming, focus of programs, isolating methods, hand health and protection techniques, improving patient’s safety and quality, training personnel) and average PCI in terms of the variables “sex, age group, education level, participants’ position, occupational field and work record (concerning work record, the criteria “improving patients` safety, and quality and isolating methods” had statistically significant differences at significant levels of 0.01 and 0.03 respectively), type of hospital and number of beds” were not statistically significant (P > 0.05). Also, the status of PCI components and average PCI are generally presented in (Table 4). According to Table 4, all PCI components and average PCI (except for isolation methods) are more than 75 % of the participants’ viewpoints.

**Table 3.** Mean and Significance Level of the Status Quo of PCI Component in Terms of Characteristics of the Subjects and the Studied Hospitals

Variables	Leadership and Programming		Focus of Programs		Isolating Methods		Hand Health and Protection Techniques		Improving Patients' Safety and Quality		Training Personnel		Average of PCI	
	Mean	P value	Mean	P value	Mean	P value	Mean	P value	Mean	P value	Mean	P value	Mean	P value
**Gender**		0.48		0.69		0.71		0.24		0.09		0.85		0.91
Male	93		81.1		67.5		88.5		79.9		90.1		79.1	
Female	91		81.9		68.8		92		75.3		91.6		78.8	
**Age group**		0.61		0.76		0.29		0.45		0.47		0.41		0.17
20-30	78.2		81.3		70.2		86.5		77.7		86.7		80.1	
30-40	75.6		80.1		65.4		87.5		76.9		90.9		77.2	
40-50	77.2		80.9		68.9		89.2		80.5		92.8		87.4	
50 <	81.7		85.9		80		90.1		77.1		66.7		83.1	
**Education**		0.91		0.51		0.62		0.98		0.42		0.57		0.58
Bachelor’s degree	92.3		77.2		80.6		67.1		88.2		91		78.4	
Master’s degree and higher	92.1		77.1		82.1		68.8		88.1		86.6		79.5	
**Participants’ position**		0.67		0.19		0.76		0.21		0.81		0.12		0.53
Manager	90.1		75.6		85.4		69.2		79.8		66.6		80.7	
Section in-charge	90.3		77.3		80.6		67.3		78.7		75.4		78.5	
Infection control expert	90.1		78		69.4		88.8		76.5		72.3		77.8	
**Occupational field**		0.03		0.12		0.56		0.76		0.53		0.25		0.61
Medicine	75.7		85.8		69.6		93.9		79.2		100		80.5	
Nursing	76.7		80.4		67.5		88		70.6		90.8		78.4	
Others	88.8		87.6		69.4		86.1		75.3		100		81.4	
**Years of work record**		0.19		0.24		0.03		0.78		0.01		0.28		0.67
< 6	78.5		82.5		73.3		90.3		82.1		100		81.3	
6-10	73.9		77.9		62.3		85.5		74.1		90.4		74.8	
11-15	76.1		80.4		64.3		87.3		74.7		95.2		76.8	
16-20	80.3		83.3		65.5		89.1		82.4		83.3		80.1	
> 20	76.8		79.8		72.3		88.6		80.8		100		79.7	
**Type of hospital**		0.1		0.2		0.1		0.1		0.1		0.4		0.21
General	74.8		78		66.9		88		77.6		62.3		78.2	
specialized	79		81.1		65.5		88.2		78.6		89.1		80.7	
**Number of beds**		0.95		0.62		0.47		0.51		0.31		0.31		0.51
< 99	76.8		80.2		67.7		88.6		79.6		88.8		78.6	
100-200	76.1		80.8		66.3		87.3		76.3		96.6		77.6	
> 200	76.5		83.2		72.7		92.4		80.1		100		80.9	

**Table 4.** Status Quo of PCI Components in the Studied Hospitals

PCI Components	Percent	SD
**Leadership and programming**	77.2	14
**Focus of programs**	80.8	13.1
**Isolating methods**	67.4	21.5
**Hand health and protection techniques**	88.2	19.1
**Improving patients**'** safety and quality**	78.8	16.2
**Training personnel**	90.3	15.3
**Total**	78.7	12.4

## 5. Discussion

 Results of the current study showed that PCI standards are in excellent condition regarding leadership and programming, focus of programs, hand health and protection techniques, improving patients` safety, and quality and training personnel in the studied hospitals and in good condition regarding isolation methods. In a similar study conducted in one of the teaching hospitals in Zanjan (North West of Iran) in 2009, the status quo of PCI standards was 82.14% for leadership and programming, 67.5% for focus of programs, 50% for isolating methods, 90% for hand health and protection techniques, 45.8% for improving patients` safety and quality, and 57.1% for training personnel (20); status of some PCI components in the mentioned research were less than the desirable limits and lower than those of the current study. These differences may be partly due to the time of the mentioned study, i.e. three years ago, because some efforts have been made to promote programs since then. That is, if a study is now conducted in the mentioned hospital, results will probably match the results of the current study. In a study conducted on some selected hospitals in Isfahan (center of Iran), the average of PCI standards was 75% (19).

According to the authors' best knowledge and the electronic search, no other study had examined all PCI components in the other hospitals of Iran. However, two studies which have pointed to one of the PCI components are listed below. In one of these studies, the leadership score (in 2010) was 46 % in Alborz Hospital, 38 % in Panzdah-E-Khordad Hospital, and 24% in emergency department of Imam Khomeini Hospital (21); these scores were lower than the scores obtained in the current study. In another study, Toorani (2010) reported that the standard “improving patients` safety and quality” scores were 78%, 63% and 54% at Shahid Hashemi Nezhad Hospital, Shahid Motahari Burn Center, and Shahid Rajai Cardiovascular Center, respectively (22). Accordingly, the status of these standards are similar in the current study and that of Shahid Hashemi Nezhad Hospital and they are significantly higher than those of the other hospitals. In their study entitled “Studying the nature of supervision structure, leadership and management in private and public hospitals”, Abor et al. showed that hospitals with quality management system were more prepared than other hospitals (23). Another study has pointed to the role and importance of leadership in hospital organization for the success of nosocomial infection control plan (24).

In the study entitled “the effect of accreditation on infection control programs”, Sekimoto et al. showed that accreditation of hospitals has a significant impact on institutionalizing infection control activities (25). Accreditation of hospitals also has a significant influence on increased patients’ satisfaction, increased quality of treatment services, decreased medical errors, decreased waiting time, observing and respecting patients’ rights at the admission time, controlling access to patients` information, and increased incentives of nurses to participate in health and treatment services. Moreover, implementing PCI standards which are among the main organization-based parts of accreditation guarantees the safety of patients, visitors and staff and decreases re-hospitalization rate, medical burden (morbidity and mortality), and economy resulted from nosocomial infections (26-28). Infection control audit is also a suitable opportunity to implement the necessary standards and changes to improve quality of services. Infection control audit causes health care workers to conform to methods and principles of controlling infection in order to prevent the spread of diseases (29, 30).

One of the strengths of the present research was that it only examined one organization-based part of accreditation standards, i.e. PCI standard components; it caused these standards to be analyzed in the form of a community. Another strength was that these standards were examined in a great number of hospitals with different features. Concerning the lack of significant difference in terms of hospital characteristics and participants’ population, it can be claimed that this study has a high internal and external reliability. Making use of managers and officials’ viewpoints in completing the questionnaires was one of the limitations of this study; participants may have exaggerated in answering the questions. To reduce the effect of this limitation, the participants were assured that analysis would not be conducted based on hospitals. Another limitation was that the insignificance found in terms of the characteristics of subjects and hospitals may be related to a small number of samples in some subgroups. The results of the current study revealed that PCI standards were significantly in appropriate condition in the studied hospitals. Thus, concerning these optimal preparation conditions in hospitals, some operational procedures must be applied to establish and implement surveillance of nosocomial infections such as reporting and recording nosocomial infection cases, infection control experts daily visits to wards and labs, strengthening follow-ups after discharge, forming infection control committee, and strengthening the intra-hospital relationships to report readmission cases and to train physicians and nurses.
